# Phenotypic and Genotypic Characterization of Enteropathogenic *Escherichia coli* (EPEC) strains in Tehran, Iran

**Published:** 2010-03

**Authors:** MR Asadi Karam, S Bouzari, M Oloomi, MM Aslani, A Jafari

**Affiliations:** 1 Department of Molecular Biology, Pasteur Institute of Iran; 2 Department of Bacteriology, Pasteur Institute of Iran

**Keywords:** Serogrouping, Pathogenicity island, Intimin, Attaching- effacing, Iran

## Abstract

**Background and Objectives:**

Enteropathogenic *Escherichia coli* (EPEC) strains can be detected by serogrouping and the presence of enterocyte attaching- effacing (*eae*) gene. Most EPEC strains belong to a certain O antigenic group. Locus of enterocyte effacement (LEE) Pathogenicity Island contains the *eae* gene and secretory proteins (ESPs) that introduce the attaching-effacing lesion. LEE inserted in tRNA genes include the *Sel*C, *Phe*U and *Phe*V sites. The aim of the present study was to genetically characterize EPEC strains isolated from children with diarrhea.

**Materials and Methods:**

Serogrouping was performed by EPEC antisera in 321 *E. coli* isolates. The presence of *ea*e, *stx, esp*B, and *eaf* genes and detection of insertion sites of LEE was done by PCR using specific primers.

**Results:**

Seventeen (5.3%) isolates belonging to 7 EPEC serogroups were identified among the whole material and all carried the *eae* gene. None of the 321 isolates showed presence of *stx* gene indicating that all 17 isolates classified as EPEC by O serogrouping did not belong to the enterohaemorrhagic *E. coli* (EHEC) group. Of these, 8 (53%) isolates carried the *eaf* and 16 (94.1%) carried the *esp*B gene. The insertion sites of LEE in serogrouped isolates were *sel*C (in 6 isolates), *phe*U (in 7 isolates) and *phe*V (in 2 isolates). The insertion site in 2 isolates was not determined by PCR.

**Conclusion:**

Serogrouping and detection of the *eae* gene showed to be reliable for detection of EPEC strains. No Shigatoxin- producing *E. coli* (STEC) strain was found among the isolates. Detection of the insertion site of LEE showed that *sel*C, *phe*U or *Phe*V are insertion sites of LEE in the EPEC strains.

## INTRODUCTION

Diarrhea is one of the important illnesses with high morbidity and mortality in children, resulting in about 1.6- 2.5 million deaths annually (1). Diarrhea is caused by a wide range of agents, including viruses, bacteria, and parasites (1). Among the bacterial pathogens, diarrheagenic *Escherichia coli* (DEC) is one of the important etiological agents of diarrhea (1, 2). Enteropathogenic *E. coli* (EPEC) strains cause large outbreaks of infantile diarrhea especially in developing countries (3, 4). According to the World Health Organization (WHO) in 1987, most EPEC strains belonged to a series of O antigenic groups known as EPEC serogroups which included O26, O55, O86, O111, O114, O119, O125, O126, O127, O128, O142 and O158 (5). Over the past 20 years, some of these serogroups, such as O26 and O11, have been assigned to other groups.

Characteristic features of EPEC strains are induction of attaching and effacing (A/E) lesions on intestinal epithelial cells, lack of enterotoxins especially SLT and lack of shigella-like invasiveness (3, 6). A/E lesion is characterized by microvilli destruction, cup-like formation, and aggregation of polarized actin and other elements at sites of bacterial attachment (5). The ability to induce A/E lesions is encoded by genes located on a 35- kb pathogenicity island (PAI) called the locus of enterocyte effacement (LEE), which contains the genes encoding *eae* (intimin), a type III secretion system, a number of secreted proteins (ESP), and the translocated intimin receptor (Tir) (2, 5). Intimin, a 94- KDa outer membrane protein encoded by the *eae* gene, is responsible for the intimate adherence between bacteria and enterocyte membranes that causes A/E lesion (2, 6). The ESP molecules (EspA, B, and D) cause formation of pores on the surface of epithelial cells that transfer effector molecules especially Tir into the host cells and disrupt the cytoskeleton to produce A/E lesion (6, 7).

Some EPEC strains have an EPEC adherence factor (EAF) plasmid that encodes the bundle- forming pili (Bfp) and the plasmid-encoded regulator (per), which is required for initial bacterial-bacterial interaction by Bfp and thus enhances virulence of EPEC strains (8–10). LEE PAI is inserted in tRNA genes. An interesting feature of the LEE is its low G+C content of 38%, which points to a lateral gene transfer from a distantly related species (11, 12). The insertion site of the LEE in the *E. coli* chromosome is different depending on the clonal phylogeny of the strains. In O157:H7 and O127:H7 serotypes, the LEE are inserted at the *sel*C locus, encoding tRNA for synthesis of selenocysteine (13–15). In O111 and O126 serogroups, the LEE is inserted at the *phe*U locus, encoding tRNA for synthesis of phenylalanine (10, 14). In some strains LEE is inserted in the *phe*V locus, encoding another tRNA for synthesis of phenylalanine (16, 17).

Reports on the prevalence of diarrheagenic *E. coli* in Iran are scarce and only some of them have used molecular methods for detection (18, 19). These studies showed that the diarrhgenic *E. coli* are the causative agent of diarrhea in Iran, but their incidence has dramatically declined (18). Studies in Iran showed that diarrheagenic *E. coli* such as EPEC and STEC strains are among the most prevalent causative agents in acute diarrhea, particularly in children (18–20). Jafari et al. reported that prevalence of STEC and EPEC strains in children with diarrhea in Tehran, Iran, was 18.9% and 12.6%, respectively (18). In study of Alikhani et al. (20), EPEC was the most prevalent pathogen (44.9%) isolated in children with diarrhea. In another report, STEC and EPEC strains were isolated in 15.5% and 6% of children with diarrhea in Tehran (21). So, knowledge of the status of the enteropathogens causing diarrhea in the Iranian population is essential for implementation of appropriate public health measures for control of the disease (18). Therefore, in this study, detection of EPEC strains among a collection of *E. coli* isolates obtained from children with diarrhea was investigated by serogrouping and the presence of the *eae* gene. Detection of the *stx* gene was performed by PCR in the *E. coli* isolates. Determination of the insertion site of LEE, and presence of the *eaf* and *esp*B genes was performed by PCR.

## MATERIALS AND METHODS

**Bacterial isolates:** A total of 321 *E. coli* isolates used in this study were obtained from children ≤5 years with diarrhea hospitalized in Tehran, Iran.

**Serogrouping of *E. coli* isolates:** Detection of EPEC strains among the 321 *E. coli* isolates was performed by O serogrouping with EPEC antisera (BioRad) by the slide agglutination method according to manufacturer's instruction.

**PCR assay:** The presence of *eae* gene was ascertained by PCR using the SK1 and SK2 primers which had been designed for the conserved region of the gene as described before (22). The list of primers and PCR conditions are given in Table 1. EPEC O127: H6 strain E2348/69 (kindly provided by Professor J.P. Nataro, Center for Vaccine development, Maryland, USA) was used as positive control.

**Table 1:** Primers and conditions for amplification of *eae*, *esp*B, *EAF* genes and *sel*C, *phe*U, *phe*V insertion sites.

Primer	Sequence ( 5' 3')	Target	Size (bp)	PCR programme	Ref
SK1	CCCGAATTCGGCACAAGCATAAGC	*eae*	863	94°C, 60 s; 45°C, 45s; 72°C, 30s^a,b^	18
SK2	CCCGGATCCGTCTCGCCAGTATTCG			94°C, 45 s; 50°C, 30s; 72°C, 30s^c,d^					
ESPB-F	GCCGTTTTTGAGAGCCAGAAT	*esp*B	633	94°C, 40s; 63°C, 45s; 72°C, 60s^a,c^	20
ESPB-R	ATCATCCTGGCCTCTGCGAAC				
EAF1	CAGGGTAAAAGAAAGATGATAA	*EAF*	399	94°C, 60s; 55°C, 45s; 72°C, 60s^a,c^	20
EAF2	TATGGGGACCATGTATTATCA				
Stx-F	GAGCGAAATAATTTATATGTG	*Stx*	518	94°C, 60s; 52°C, 60s; 72°C, 60s^a,c^	19
Stx-R	TGATGATGGCAATTCAGTAT				
K260	GAGCGAATATTCCGATATCTGGTT	*sel*C	527	94°C, 60s; 60°C, 45s; 72°C, 45s^a,c^	16
K261	CCTGCAAATAAACACGGCGCAT				
K913	CATCGGCTGGCGGAAGATAT	*phe*U	300	94°C, 45s; 55°C, 45s; 72°C, 30s^a,c^	16
K914	CGCTTAAATCGTGGCGTC				
pheV-F	CTGGGTATTGCGGTATCGGTGA	*phe*V	604	94°C, 40s; 55°C, 45s; 72°C, 30s^a,c^	this study
pheV-R	GCTGGAGTTTGGACGGGGGTAA				

a before the first cycle the sample was denatured for 5 min at 94°C.

b five cycles.

c After the last cycle, the sample was extended for 5 min at 72°C.

d Twenty- five cycles

The presence of the *stx* gene was ascertained in all *E. coli* isolates by PCR using primers designed by Toma *et al*. (23) (Table 1). Shigatoxin producing *E. coli* (STEC) O157 was used as reference strain. A pair of primers designed by Kobayashi *et al*. (24) for the conserved region of *esp*B was used to ascertain the presence of the gene (Table 1). EPEC O127:H6 strain E2348/69 was used as the reference strain. The presence of *eaf* gene was investigated among the isolates by PCR using EAF1 and EAF2 primers designed by Kobayashi *et al*. (24) (Table 1). EPEC O127:H6 strain E2348/69 was used as reference strain. The insertion site of the LEE PAI was determined by PCR using K260, K261 and K913, K914 primers for *sel*C and *phe*U sites, respectively (16). Primers for *phe*V site were designed in the present study (Table 1). Insertion in any of these sites would result in negative amplification.

## RESULTS

In this study, 321 *Escherichia coli* isolates were collected from children ≤5 years with diarrhea hospitalized in Tehran, Iran. The isolates were confirmed as *E. coli* by culture and biochemical properties obtained by the API kit.

The results of O serogrouping by EPEC antisera showed that 17 (5.3%) isolates of the 321 *E. coli* isolates were typeable with used antisera. These 17 isolates belonged to 7 serogroups, namely O127 (6 isolates), O86 (5 isolates), O126 (2 isolates), O142 (1 isolate), O55 (1 isolate), O119 (1 isolate) and O128 (1 isolate).

Seventeen (5.3%) isolates harbored the *eae* gene and all serogrouped by EPEC antisera and none carried the *stx* gene in the 321 *E. coli* isolates, thus among the *E. coli* isolates, none of them classified as STEC. PCR results showed that 8 (47%) isolates of 17 serogrouped isolates carried the *eaf* plasmid gene. Gene for *esp*B was detected among 16 (94.1%) isolates and 16 (94.1%) isolates produced an amplicon with espB primers. Among the isolates, *sel* C was the insertion site for 6 isolates (35.3%), *phe* U for 7 isolates (41.1%) and *phe*V for 2 isolates (11.8%). Insertion site of 2 isolates (11.8%) was not detectable with these primers (Table 2).

**Table 2:** Insertion sites present in EPEC isolates

Insertion sites	No. Isolates positive
*sel*C-/*phe*U+/*phe*V+	6
*sel*C+/*phe*U-/*phe*V+	7
*sel*C+/*phe*U+/*phe*V-	2
*sel*C-/*phe*U-/*phe*V+	2
**Total**	**17**

(−) Disrupted site (insertion) (+) Intact site (no insertion)

## DISCUSSION

Phenotypic assays such as serogrouping with traditional antiserum are the routine methods that have been widely used in clinical laboratories (1, 25). Serogrouping has been shown to be insufficient for identification of true EPEC strains mainly because somatic (O antigenic) markers sometimes do not correlate with pathogenicity of an isolate (25, 26). For example, Giammanco *et al*. (27) showed the prevalence of pathogenic genes in serogrouped EPEC strains was 75% and Bouzari *et al*. (9) showed that only 65% of serogrouped EPEC strains carried virulence- associated genes. Thus, molecular methods using primers targeting specific virulence genes of the isolates offers a reliable approach to detect EPEC strains from patients or patient samples (25, 26). Furthermore, these methods normally yield rapid results with a high sensitivity and specificity (1). In our study the results of PCR detection of *eae* gene showed that all 17 isolates identified as EPEC by serogrouping also harbored the *eae* gene indicating the usefulness of the serogrouping detection of EPEC strains in this instance. Many of shigatoxin producing strains of *E. coli* (STEC) also harbor the *eae* gene (2, 6). Presence of *stx* gene can distinguish STEC strains from EPEC strains (2, 5, and 6). In our study, none of the *E. coli* isolates carried the *stx* gene, thus we did not find any STEC strains, similar to results obtained from Taiwan and Tanzania (25, 1). Epidemiologically, despite large outbreaks of infant diarrhea due to EPEC in industrialized countries in the past (28), EPEC strains still remain a major cause of mortality in infants in developing countries (4, 8, 28). The prevalence of EPEC in our study (5.3%) was less than another report (8.4%) from Iran (19). This difference could be due to differences in the technical handling of sampls and methods used for isolation of the bacteria. The prevalence of EPEC reported from other countries is different: Tanzania (4.6%) (1), Thailand (5.5%) (29), Brazil (34%) (30) and Switzerland (16%) (31). In our isolates, O127 was the most frequent serogroup. The results of serogrouping in different countries suggest that the rate of EPEC strains in each of the EPEC serogroups vary (4, 32). Our results showed that the prevalence of EPEC strains was higher than STEC strains, but in another report in Iran (19), the isolation rate of STEC strains was higher than that of EPEC (44.7% and 8.4%, respectively). Similar to our results, the prevalence rate of EPEC reported from Brazil (32), Taiwan (25) and Tanzania (1) was higher than STEC strains. It is possible that EPEC infections in early childhood confer cross-reactive protective immunity against STEC strains that have similar antigens such as *eae* with EPEC strains and this could explain why human infections with *eae* positive STEC occur less frequently in developing countries (32). LEE PAI inserted in tRNA genes, including *sel*C, *phe*U and *phe*V insertion sites (11, 12). Our results showed that *sel*C, *phe*U and *phe*V are present among EPEC strains. Bertin *et al*. (15) showed that the LEE was inserted at *phe*V (6 isolates), *phe*U (2 isolates) and selC sites (3 isolates). In our study, *sel*C and *phe*U were predominant sites for insertion of LEE. The insertion site for 2 isolates was not detected because both *phe*U and *sel*C sites were disrupted in these isolates. *Esp*B gene is the second gene in LEE, after the eae gene that is necessary for induction of A/E lesions (6, 7). Thus, presence of *esp*B can confirm the presence of LEE and eae gene. In our study, only one eae-positive isolate lacked *esp*B gene. This discrepancy between the presence of *eae* and *esp*B genes in this isolate was not further investigated although the PCR reaction was several times repeated to make sure of its reproducibility.

Overall*,* in the present study, serogrouping and PCR amplification of *eae* gene revealed similar results in detection of EPEC strains, although genotyping of isolates could be more informative and conclusive.
